# Associations of Insecticide Exposure with Childhood Asthma and Wheezing: A Population-Based Cross-Sectional Study in Sanya, China

**DOI:** 10.3390/toxics12060392

**Published:** 2024-05-27

**Authors:** Yabin Hu, Guiyan Yang, Dan Wang, Wangyang Gu, Dan Xie, Tingyue Huang, Peng Xue, Jingyi Tang, Hui Wei, Shenghui Li, Shilu Tong, Shijian Liu

**Affiliations:** 1 Hainan Branch, Shanghai Children’s Medical Center, School of Medicine, Shanghai Jiao Tong University, Sanya 572022, China; yabinhu@126.com (Y.H.); wangyanggu52@163.com (W.G.); 13609897638@163.com (T.H.); 88255852@163.com (H.W.); 2 Department of Clinical Epidemiology and Biostatistics, Shanghai Children’s Medical Center, School of Medicine, Shanghai Jiao Tong University, Shanghai 200127, China; xuepeng992021@163.com (P.X.); tangjingyi1117@163.com (J.T.); 3 School of Public Health, Shanghai Jiao Tong University, Shanghai 200025, China; lsh9907@163.com; 4 National Institute of Environmental Health, Chinese Centers for Disease Control and Prevention, Beijing 102206, China; tongshilu@nieh.chinacdc.cn; 5 School of Public Health and Social Work, Queensland University of Technology, Brisbane 4001, Australia

**Keywords:** asthma, childhood, insecticide, wheezing

## Abstract

Insecticide exposure may affect childhood asthma/wheezing, but evidence is scarce in low- and middle-income countries. We conducted a population-based cross-sectional study in Sanya, China. Generalized linear models were adopted to assess the associations of insecticide exposure with childhood asthma/wheezing, reported as odds ratios (ORs) and 95% confidence intervals (CIs). A subgroup analysis was performed to explore the possible effects of sociodemographic and environmental factors on these associations. The median age of the 9754 children was 6.7 years, and 5345 (54.8%) were boys. The prevalences of ever asthma (EA), ever wheezing (EW), and current wheezing (CW) were 7.4%, 5.3%, and 2.9%, respectively. We found a greater prevalence of childhood EA with insecticide exposure (OR = 1.18, 95% CI: 1.00, 1.38). Outdoor insecticide exposure was associated with elevated ORs for EA (1.24, 95% CI: 1.03, 1.50), EW (1.27, 95% CI: 1.03, 1.57), and CW (1.38, 95% CI: 1.04, 1.81). The *p* for the trend in insecticide exposure frequency was significant for EA (*p* = 0.001) and CW (*p* = 0.034). These adverse impacts were pronounced in girls who were exposed to low temperatures. Our findings suggest adverse effects of insecticide use, especially outdoors, on childhood asthma/wheezing. Further studies are warranted to verify this association and develop tailored prevention measures.

## 1. Introduction

Asthma is the most prevalent noncommunicable chronic respiratory disease, affecting 262.4 million people worldwide, with 37.0 million new cases emerging in 2019. Low- and middle-income countries (LMICs) are responsible for 84% of disability-adjusted life years (DALYs) and 96% of asthma-related deaths [1,2]. While asthma can occur at any age, the majority of patients first experience symptoms in childhood [3,4,5], with the highest incidence occurring in the 1–4 year age group worldwide in 2019 [1,2]. Childhood asthma poses a great threat to families, society, and healthcare systems, imposing considerable public health and economic burdens [2,3,6,7,8].

The prevalence of childhood asthma continues to rise in many countries and regions [3,4,5]. Over the course of 27 years (1993–2020), the Global Asthma Network’s Phase I research has revealed a considerable increase in the percentage point prevalence of ever-existing asthma (1.25%) among 13- to 14-year-old children globally. The prevalence of ever-existing asthma (ever asthma) increased in upper-middle-income countries (1.19% for 6- to 7-year-old children and 1.91% for 13- to 14-year-old children) and high-income countries (2.07% for 6- to 7-year-old children and 2.79% for 13- to 14-year-old children). The prevalence of current wheezing increased in lower-middle-income countries (1.99% for 6- to 7-year-old children and 1.69% for 13- to 14-year-old children) [6]. The increasing prevalence of childhood asthma and wheezing is expected to continue as many transitional communities adopt lifestyles similar to those of high-income countries and rapidly urbanize. Yi et al. [9] reported that the incidence and prevalence of asthma in children and adolescents aged 1–19 years in 2019 were 0.7% (95% UI: 0.5–1.1%) and 2.9% (95% UI: 1.9–4.4%), which increased by 3.3% and 0.6%, respectively, during the 1990–2019 period according to data from the 2019 Global Burden of Disease Study for China. In some high-income cities, such as Shanghai, there was a sharp increase in the prevalence of asthma among 3- to 7-year-old children in which the overall prevalence increased by more than sixfold, growing from 2.1% in 1990 to 14.6% in 2019 [10].

It is necessary to gain a better understanding of the causal factors of childhood asthma and to develop tailored prevention measures that are effective in lessening the global burden of childhood asthma. Many factors lead to the occurrence and exacerbation of childhood asthma, including genetics and environmental factors [11]. The increase in childhood asthma prevalence in recent decades cannot be attributed solely to genetic alterations. Therefore, environmental exposure is an important contributor to this increase [3,11]. Many studies in the literature have provided compelling evidence that nonoptimal meteorological factors and air pollution are triggers for the development and exacerbation of childhood asthma [12,13,14,15,16,17,18,19,20,21,22,23,24,25].

Recently, increasing attention has been given to insecticide exposure and childhood respiratory and allergic diseases such as asthma [26,27,28,29,30]. Mora and colleagues suggested that prenatal exposure to ethylenethiourea (ETU, a urinary pesticide metabolite) was associated with respiratory outcomes in the first year of life [27]. Maritano et al. [26] reported that compared with a lack of pesticide use, there was a positive association between infant wheezing and pesticide use in both trimesters (odds ratio, OR = 1.72; 95% confidence interval, CI = 1.11–2.65). Raanan and colleagues showed that early-life exposure to organophosphate pesticides (OPs) was associated with childhood respiratory symptoms in Salinas Valley, an agricultural community in California [28]. However, some studies have reported no associations between exposure to insecticides during pregnancy or household pesticide use in early life and childhood asthma [29,30].

Therefore, the association between childhood asthma and exposure to insecticides is inconclusive. Moreover, since the majority of previous studies were conducted in high-income countries, there is a paucity of evidence from LMICs. In view of these knowledge gaps, we applied a population-based observational study involving preschool and school children aged 2–11 years in Sanya, a tropical city in Hainan Province, China, to (i) evaluate insecticide exposure and childhood asthma and wheezing and (ii) further determine the vulnerable population among children to provide evidence for developing tailored strategies to better prevent and manage childhood asthma and wheezing.

## 2. Materials and Methods

### 2.1. Study Site and Population

**Figure 1:**
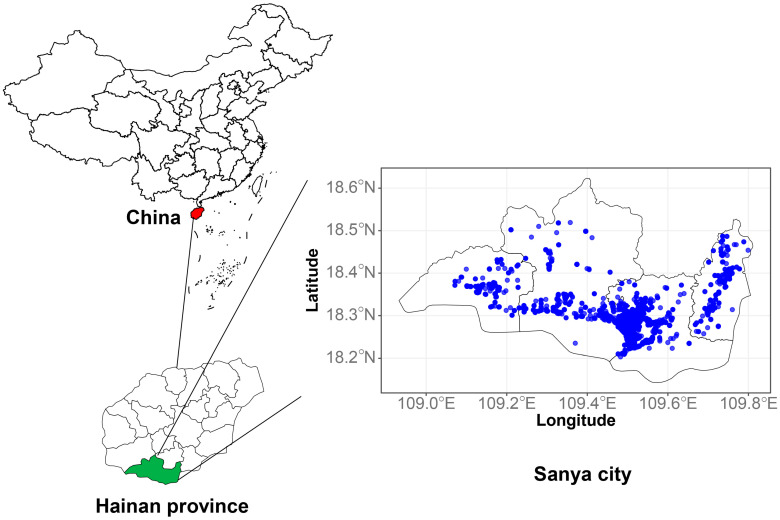
The geographical distribution of the study location and the participants’ residential addresses.

This was wave I of the Sanya Children Cohort Study in Hainan Province, China, a population-based cross-sectional study which was carried out from 31 October to 19 December 2022 (Figure 1). Sanya City is located in southernmost China, with coordinates of N 18°09′34″–18°37′27″ and E 108°56′30″–109°48′28″. At low latitudes, Sanya has a tropical oceanic monsoon climate, the majority of Sanya City is in suburban or rural areas, and fruit cultivation is common. By the end of 2022, the mean population density was 381 km.

We used a stratified-cluster randomized sampling approach to identify eligible children from 197 kindergartens and 116 primary schools in four districts. Finally, 20 kindergartens and 13 primary schools were randomly selected. We first explained the purpose and content of the project to the principals and teachers in advance. Then, the teachers conveyed these messages to the children’s legal guardians and assisted them with filling out the e-questionnaire on their mobile phones. A total of 11,318 children participated in our study initially, and 10,305 (response rate: 91.0%) completed the questionnaires through their guardians. After excluding those aged <2 years or >12 years or those whose data contained age-filling errors (N = 551), we included 9754 children aged 2–11 years from the final analysis.

Ethical approval for this study was obtained from the Ethics Committee of Sanya Women and Children’s Hospital (ID: SYFYIRB20220063). All legal guardians provided informed consent prior to data collection.

### 2.2. Outcomes

We adopted a self-administered e-questionnaire based on the International Study of Asthma and Allergies in Childhood [31], the Swedish Dampness in Buildings and Health Study [32], and the China, Children, Homes, Health Study [33] to collect data on health outcomes, exposures, and covariates. In this study, the outcomes included childhood asthma and wheezing, which were defined below as follows:

(i) Ever asthma: Has your child ever been diagnosed with asthma by a doctor?
(ii) Ever wheezing: Has your child ever experienced wheezing, whistling, or dyspnea?
(iii) Current wheezing: Has your child had wheezing, whistling, or dyspnea symptoms in the past 12 months?

### 2.3. Main Exposure

In this study, the main form of exposure was the use of insecticides, and we collected the following information:

(i) Exposure or not: Have you used insecticide in the past 12 months? Yes or no;
(ii) If yes, where have you used insecticide? Outdoor, indoor, or for pets;
(iii) If yes, what frequency do you use insecticides? Only once, once per half year, once per quarter, once per month, or once per week.

In this study, insecticide exposure was defined as a frequency of insecticide use of more than once in the past 12 months, including both indoor and outdoor use.

### 2.4. Covariates

We collected information on sociodemographic characteristics and prenatal and early life exposure data using a questionnaire. Each child’s age was calculated as the date of the survey minus their date of birth; each child’s sex was male or female; the household income per month (in Chinese Yuan, CNY) was <3000, 3000–5999, 6000–8999, 9000–11,999, or >12,000; and the number of maternal gestational weeks was <37, 37–<42, or ≥42; the questionnaire also collected data on the presence or absence of miscarriage; family history of allergy (asthma, allergic rhinitis, atopic dermatitis, food allergy, drug allergy, etc.); exposure to antibiotic use during pregnancy or within the first year of the child’s life; decoration and passive smoking in early life; the presence of a pet since conception; the presence of dampness/mold; the traffic conditions within 50 m of the child’s house (much, not much, and less); and the frequency of park visits (≤once/month, 2–3 times/month, 1–2 times/week, 3–5 times/week, and ≥once/day). The missing values were defined as an unknown group. The number of missing values was 51 for gestational weeks, 870 for decoration, 879 for dampness/mold, and 870 for the frequency of park visits.

Information on the annual average ambient temperature and relative humidity for Sanya from 2020 to 2022 within 1 km buffers was extracted from the National Meteorological Science Data Center “http://data.cma.cn/ (accessed on 10 April 2023)”. Exposures were then matched against the geocoded participants’ current residential addresses. There were no missing values for these two indices.

### 2.5. Statistical Analysis

The statistical analysis procedures were mainly classified into three steps. Step I comprised a descriptive analysis for outcomes, exposures, and covariates. Continuous data (e.g., the child’s age) were presented as median and interquartile range (IQR) values, and the Mann–Whitney U test was used to compare the differences between two groups. Categorical data were presented as numbers and percentages, and Pearson’s chi-squared test or Fisher’s exact test was used to compare differences among groups. In Step II, univariable and multivariable binomial generalized linear models (GLMs) with a logit link were conducted to assess the associations of insecticide exposure with childhood asthma and wheezing, reported as ORs and 95% CIs. In this step, to construct appropriate multivariable models, we comprehensively considered the previous literature, the results of single-variable models, the Akaike information criterion (AIC), and the directed acyclic graph (DAG, Figure S1) drawn using DAGitty v3.1 software [34]. In Model II, we adjusted for sociodemographic factors (i.e., the child’s age, the child’s sex, household income, gestational week, history of miscarriage, and family history of allergy). In Model III, we further controlled for environmental exposures (i.e., antibiotics, passive smoking, traffic within 50 m, decoration, having a pet, mold, the frequency of park visits, ambient temperature, and relative humidity).The variance inflation factor (VIF) was calculated in the adjusted models to avoid multicollinearity (VIF < 5). In addition, we calculated the *p*-value for the trend in exposure frequency to insecticides and childhood asthma/wheezing assigning “0, 1, 2, 3, 4, and 5” to “no exposure, only once, once per half year, once per quarter, once per month, and once per week”. In Step III, a stratified analysis was performed to explore the possible effects of sociodemographic and environmental variables on the insecticide–asthma/wheezing association. The Z test was utilized to evaluate the differences among subgroups [35].

A sensitivity analysis was carried out to verify the robustness of the final adjusted models, and the main results were (i) adjusted for different potential confounders in the GLM; and (ii) mixed-effect regression models (MERMs) were run with districts as random effects.

All analyses were performed using the “bruceR”, “tydiverse”, “gtsummary”, “forestmodel”, “ggplot2”, “lme4”, and “car” packages in R 4.2.2 (R Core Team). A two-tailed *p* < 0.05 was considered to indicate statistical significance.

## 3. Results

### 3.1. Basic Characteristics

Figure 1 shows the study location and the participants’ residences. Among the 9754 children, the median age (IQR) was 6.7 (2.9) years, and 5345 (54.8%) were boys. The percentages of patients who experienced ever asthma (EA), ever wheezing (EW), and current wheezing (CW) were 726 (7.4%), 520 (5.3%), and 283 (2.9%), respectively. Table 1 presents the basic characteristics of the children with and without asthma/wheezing. The median ambient temperature and relative humidity were 26.1 °C and 82.3%, respectively. The prevalence of EA (8.5% vs. 6.2%, *p* < 0.001), EW (6.3% vs. 4.2%, *p* < 0.001), and CW (3.5% vs. 2.2%, *p* < 0.001) was greater in boys than in girls. The proportions of children with EA, EW, and CW were greater than those without EA, EW, and CW in the following groups: preterm birth, having a history of miscarriage, having a family history of allergy, using antibiotics during pregnancy or within the first year of the child’s life, having a pet since conception, exposure to passive smoking, decoration, insecticide, dampness/mold, the presence of much traffic within 50 m of the house, and not frequently visiting parks (≤once/month).

### 3.2. Association of Insecticide Exposure with Asthma/Wheezing in Children

**Figure 2:**
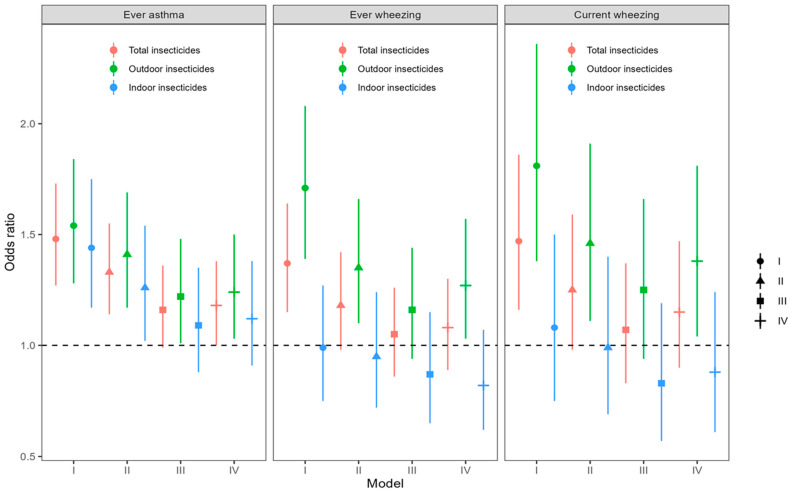
Exposure to insecticides and odds ratios of childhood asthma/wheezing. Model I: crude model; Model II: adjustment for child’s age, child’s sex, household income, gestational week, miscarriage status, and family history of allergy; Model III: based on Model II, further controlling for exposure to antibiotics, passive smoking, traffic within 50 m, decoration, having a pet, mold, frequency of park visits, ambient temperature, and relative humidity; Model IV: model with lowest Akaike information criterion in which insecticide exposure, child’s sex, gestational week, family history of allergy, antibiotic use during pregnancy, antibiotic use within child’s first year, exposure to passive smoking, mold, frequency of park visits, and ambient temperature were included.

Figure 2 depicts the OR of childhood asthma/wheezing associated with insecticide exposure. According to Model I (univariable/crude model), exposure to total insecticides was associated with an increased OR for EA (1.48, 95% CI: 1.27, 1.73), EW (1.37, 95% CI: 1.15, 1.64), and CW (1.47, 95% CI: 1.16, 1.86). Outdoor insecticide exposure was associated with elevated ORs for EA (1.54, 95% CI: 1.28, 1.84), EW (1.71, 95% CI: 1.39, 2.08), and CW (1.81, 95% CI: 1.38, 2.36). Exposure to indoor insecticides was only positively associated with EA (OR = 1.44, 95% CI: 1.17, 1.75).

In Model II, after adjusting for the child’s age, child’s sex, income, gestational week, miscarriage status, and family history of allergy, we observed a positive association of outdoor insecticide exposure with childhood EA (OR = 1.41, 95% CI: 1.17, 1.69), EW (OR = 1.35, 95% CI: 1.10, 1.66), and CW (OR = 1.46, 95% CI: 1.11, 1.91), respectively. In addition, total and indoor insecticide exposures were associated with increased ORs for EA (1.33, 95% CI: 1.14, 1.55, and 1.26, 95% CI: 1.02, 1.54, respectively).

In Model III, outdoor insecticide exposure was associated with an increased OR for EA (1.22, 95% CI: 1.01, 1.48) after further controlling for exposure to antibiotics, passive smoking, traffic conditions within 50 m of the house, decoration, pet ownership, mold, frequency of park visits, ambient temperature, and relative humidity.

According to the lowest Akaike information criterion, the child’s sex, gestational week, family history of allergy, antibiotic use during pregnancy, antibiotic use within the first year of the child’s life, exposure to passive smoking, mold, frequency of park visits, insecticides, and ambient temperature were incorporated for adjustment in Model IV. Moreover, outdoor insecticide exposure was still associated with elevated ORs for EA (1.24, 95% CI: 1.03, 1.50), EW (1.27, 95% CI: 1.03, 1.57) and CW (1.38, 95% CI: 1.04, 1.81). Total insecticide exposure was associated with an elevated OR for EA (1.18, 95% CI: 1.00, 1.38).

Table S1 illustrates the values of the variance inflation factor (VIF) in the final adjusted models. This indicates that there was no multicollinearity issue in which all VIFs were <2. Table S2 presents a comparison of the results of the GLM with a logit link and the MERM with districts as random effects. The GLM results presented in the main text were almost the same as those of the MERM.

Table 2 shows the associations of the frequency of exposure to insecticides with asthma/wheezing in children. A higher frequency of insecticide exposure (once/week) was associated a greater ORs of childhood EA (2.51, 95% CI: 1.73, 3.56), EW (1.87, 95% CI: 1.16, 2.88), and CW (2.24, 95% CI: 1.22, 3.79) in the crude model. In addition, we calculated the *p* for the trend and found statistical significance for EA (*p* = 0.001) and CW (*p* = 0.034) in the adjusted model. This indicates that the greater the frequency of insecticide exposure is, the greater the risk of childhood asthma and wheezing.

### 3.3. Effect Modification

**Figure 3:**
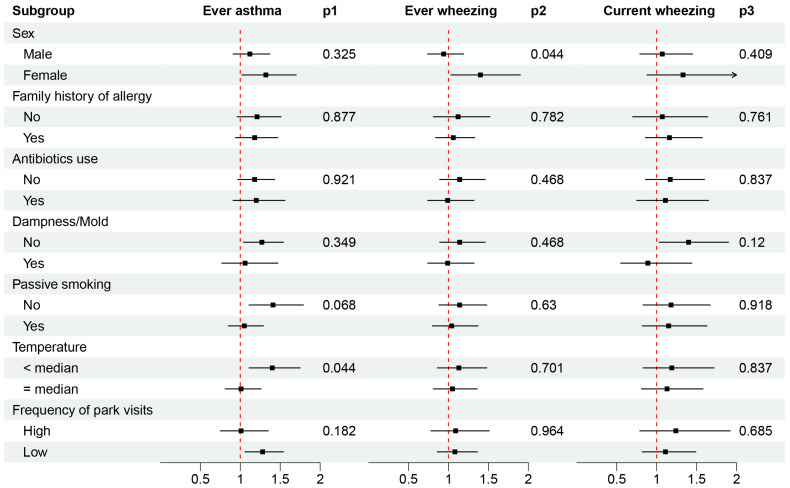
Effect modification on the insecticide–asthma/wheezing association by sociodemographic and environmental factors. The data are presented as odds ratios and 95% confidence intervals. In the adjusted models, the child’s sex, family history of allergy, antibiotic use during pregnancy or within the first year of the child’s life, exposure to passive smoking, mold, frequency of park visits, and ambient temperature were controlled. *p*-values (*p*1, *p*2, and *p*3) were calculated using the Z test to test for differences between subgroups; high frequency of park visits: ≥once/week; low frequency of park visits: <once/week.

Figure 3 demonstrates the effect modification on the insecticide–asthma/wheezing association according to sociodemographic characteristics and environmental factors. Exposure to insecticides was associated with elevated ORs for EA (1.32, 95% CI: 1.02, 1.70) and EW (1.40, 95% CI: 1.03, 1.90) in girls. A null association was found in boys (1.12, 95% CI: 0.91, 1.37, and 0.94, 95% CI: 0.74, 1.19, respectively. An insecticide–EA association was detected in the following subgroups: without dampness/mold exposure (OR = 1.27, 95% CI: 1.04, 1.54), without passive smoking (OR = 1.41, 95% CI: 1.11, 1.79), at a low temperature (<median) (OR = 1.40, 95% CI: 1.11, 1.75), and with a low frequency of park visits (<once/week) (OR = 1.28, 95% CI: 1.06, 1.54).

## 4. Discussion

### 4.1. Key Findings

In this population-based cross-sectional study in Sanya, China, we found an independent adverse effect of insecticide use on asthma and wheezing among preschool and primary school children aged 2–11 years. Furthermore, outdoor insecticide use was associated with elevated odds of asthma and wheezing in children, while a null association was observed indoors. In addition, the greater the frequency of insecticide exposure, the greater the risk of asthma and wheezing. These detrimental effects were pronounced in girls and in children exposed to low temperatures.

### 4.2. Comparison with Other Studies

To the best of our knowledge, this is the first population-based study concerning the associations of insecticide exposure with asthma and wheezing among preschool and primary school children in China. The climate of Sanya is characterized by a tropical oceanic monsoon climate with an average annual temperature of 26.0 °C and an average annual relative humidity of 82.4%. These climate conditions are suitable for the proliferation of insects, especially mosquitoes, resulting in a high number of mosquitoes each year and a relatively high rate of insecticide use (38.0% in this study). Therefore, it is important to examine whether insecticide exposure in early life is associated with increased risks of childhood asthma and wheezing. In this study, the prevalences of ever having asthma, ever having wheezing, and currently having wheezing were 7.4%, 5.3%, and 2.9%, respectively, which are lower than our previous findings of 13.9–20.2% in Shanghai among children of similar ages [10,36,37]. Disparities in the economy, urbanization, and air pollution may partially play a role.

Mounting epidemiological evidence indicates that prenatal exposure to insecticides [26,27,38,39] and early childhood exposure [40] or both [28,41] are associated with an increased risk of childhood asthma and wheezing, but most of these studies were carried out in high-income countries. Mora et al. [27] reported that infants whose mothers had higher ETU concentrations during late pregnancy had a reduced risk of wheezing. In the same study, Islam and colleagues observed no association between prenatal pesticide exposure and asthma/wheezing, though they found that current exposure to pyrethroid insecticides might contribute to asthma among 5-year-old children [41]. Consistent with their findings, we observed an adverse effect of overall exposure to insecticides on asthma and wheezing in children aged 2–11 years. Perla et al. [40] reported no association between dialkylphosphate (DAP) and an increased risk of asthma and wheezing in school-aged children, but dichlorodiphenyldichloroethylene (DDE) had an adverse effect on current wheezing. Xiao and colleagues observed a null association between household pesticide use and current asthma among 41,423 American children during the 2017–2018 period [30]. They found that children who were exposed to household mold had a greater prevalence of current asthma (10.8%) than did those who were not exposed (7.2%), and children with household mold exposure had 1.41 times (95% CI: 1.07, 1.87) greater odds of having current asthma. The association was pronounced among boys (OR = 1.57, 95% CI: 1.03, 2.38) but not girls (OR = 1.28, 0.90, 1.83, *p* < 0.001 for interaction). In this study, we found that either insecticide or dampness/mold exposure was independently associated with elevated odds of childhood asthma and wheezing. The differences among studies are possibly related to study designs (e.g., cross-sectional vs. prospective cohort), study participants (e.g., different age groups and races/ethnicities), study locations (e.g., different countries and cities), assessments of insecticide exposure (e.g., urine/serum detection vs. questionnaires), windows of exposure (e.g., prenatal vs. postnatal), and types of exposure (e.g., overall vs. specific metabolites).

Thus, previous findings on the insecticide–asthma/wheezing association are inconsistent. Our findings provide some supporting evidence of the adverse impacts of current insecticide exposure on childhood asthma and wheezing. We also observed that some other adverse exposures (e.g., passive smoking and antibiotic use) were associated with childhood asthma and wheezing, which is consistent with previous studies [42,43,44]. We performed additional analyses to assess the impacts of air pollution (O_3_ and PM_2.5_) and greenness on childhood asthma and wheezing but found no apparent associations in Sanya, where the average annual concentrations of air pollution are relatively low. Sanya is one of the cities with the lowest air quality indices in China [45]. Our previous studies in Shanghai showed an adverse effect of air pollution and a protective influence of greenness on childhood asthma [16,18,46,47]. There is little evidence of which and how sociodemographic and environmental factors modify the insecticide–asthma/wheezing link. We observed that this association was more pronounced for girls, children exposed to low temperatures (<median value), and those with a low frequency of park visits (<once/week).

### 4.3. Potential Mechanisms

The mechanisms underlying the relationships between insecticide exposure and childhood asthma/wheezing have not been fully elucidated. Several potential mechanisms involving inflammatory responses, oxidative stress, cytokine imbalance, immunosuppression, immune dysregulation, and endocrine disruption have been proposed [38,48]. Duramad et al. [49] reported that mothers employed in agricultural regions with relatively high levels of OP usage were more likely to give birth to infants with elevated T helper 2 (Th2) cytokines, which are associated with the development of asthma, as well as a diagnosis of wheezing at the age of two years. Animal experiments and in vitro studies have provided evidence of the potential mechanisms through which chlorpyrifos, a pyrethroid insecticide, may affect respiratory outcomes. In guinea pigs, chlorpyrifos was found to induce airway hyperactivity by causing the dysfunction of neuronal M2 receptors, thereby impacting asthma [50]. Additionally, an in vitro study demonstrated that chlorpyrifos inhibited the proinflammatory function of macrophages, suggesting that insecticide-induced immunosuppression might contribute to lower respiratory tract infections [51].

Notably, outdoor insecticide exposure was linked to increased odds of childhood asthma and wheezing in this study. This is probably because there are fewer preventive actions, such as wearing masks, in an outdoor environment with insecticide use. In addition, more outdoor activities cause a child to breathe quickly and deeply, resulting in higher levels of exposure. Because indoor/outdoor insecticide exposure was evaluated using questionnaire questions about “indoor/outdoor insecticide use” in this study, it is an external exposure measure with a degree of bias. Further studies involving the precise detection of insecticides and their metabolites using an HPLC-MS/MS method are warranted to better understand the specific underlying mechanisms [52].

### 4.4. Strengths and Limitations

Our study has three notable strengths. To our knowledge, this is the first population-based study focusing on the effects of insecticide exposure on childhood asthma and wheezing in China. Furthermore, representative samples were obtained through a stringent multi-strata random sampling approach. The large sample size and high response rate (91.0%) in this study provided ample statistical power to assess the associations effectively. In addition, our study included as much information on sociodemographic and environmental factors as possible, which enabled us to minimize the impact of potential confounders and explore potential effect modifiers.

Several major limitations of this study should also be acknowledged. As the first wave of a prospective cohort study, this research is cross-sectional in nature, so causation could not be established. In the foreseeable future, we will conduct a series of follow-up studies to identify causal relationships. All data on outcomes, insecticide exposure, and covariates were gathered through questionnaires, without actual medical record confirmations of outcomes or exact detections of insecticide exposure; thus, an information bias exists to some extent. There is no doubt that recall bias cannot be ignored due to the nature of this survey, especially during the COVID-19 pandemic. However, the questionnaire used in this study was based on our previous studies in Shanghai, and its validity and reliability were quite high [10,36,37]. Additionally, the dose of indoor or outdoor insecticide exposure or both and the types of insecticides used were not distinguished. Further research on specific kinds of insecticides and childhood asthma/wheezing is necessary to elucidate such links and formulate effective preventive strategies.

### 4.5. Future Directions

To better understand the insecticide–asthma/wheezing connection, subsequent studies should focus on (i) more relevant studies across different regions and countries, especially in LMICs; (ii) more precise detection methods and specific kinds of insecticides and their links with childhood asthma/wheezing; (iii) well-designed longitudinal studies involving various insecticide exposures across the lifespan; and (iv) the interactive effects of insecticides and air pollution/meteorological factors on childhood asthma and wheezing [53]. All of these findings may help to identify the predominant types of insecticides and critical exposure windows associated with childhood asthma/wheezing and contribute to the exploration of potential mechanisms that could explain this association.

## 5. Conclusions

Our findings suggest adverse effects of self-reported insecticide use, especially outdoors, on childhood asthma and wheezing. The greater the frequency of insecticide exposure is, the greater the likelihood of asthma and wheezing in children. Such detrimental effects were stronger in girls and in children exposed to low temperatures. Further research with precise exposure data and specific kinds of insecticides is warranted to verify such associations, unveil potential underlying mechanisms, build a strong body of evidence to support legislative decision-making regarding insecticide use, and develop effective, tailored prevention measures to curb the global burden of childhood asthma.

## Figures and Tables

**Table 1:** The basic characteristics of participants with and without asthma and wheezing.

Characteristics	Overall, N = 9754	Ever Asthma		*p*-Value ^1^	Ever Wheezing		*p*-Value ^1^	Current Wheezing		*p*-Value ^1^
		No, N = 9028	Yes, N = 726		No, N = 9234	Yes, N = 520		No, N = 9471	Yes, N = 283	
Child age, years, median (IQR)	6.7 (2.9)	6.7 (2.9)	6.7 (3.0)	0.600	6.7 (2.9)	7.1 (2.7)	<0.001	6.7 (2.9)	6.6 (3.0)	0.700
Sex, n (%)				<0.001			<0.001			<0.001
Male	5345 (54.8)	4893 (91.5)	452 (8.5)		5010 (93.7)	335 (6.3)		5158 (96.5)	187 (3.5)	
Female	4409 (45.2)	4135 (93.8)	274 (6.2)		4224 (95.8)	185 (4.2)		4313 (97.8)	96 (2.2)	
Household income per month, CNY, n (%)				0.038			<0.001			0.003
<3000	2932 (30.1)	2689 (91.7)	243 (8.3)		2817 (96.1)	115 (3.9)		2854 (97.3)	78 (2.7)	
3000–5999	2782 (28.5)	2578 (92.7)	204 (7.3)		2645 (95.1)	137 (4.9)		2721 (97.8)	61 (2.2)	
6000–8999	1586 (16.3)	1459 (92.0)	127 (8.0)		1491 (94.0)	95 (6.0)		1537 (96.9)	49 (3.1)	
9000–11,999	1022 (10.5)	953 (93.2)	69 (6.8)		945 (92.5)	77 (7.5)		977 (95.6)	45 (4.4)	
>12,000	1432 (14.7)	1349 (94.2)	83 (5.8)		1336 (93.3)	96 (6.7)		1382 (96.5)	50 (3.5)	
Gestational week, weeks, n (%)				0.005			0.002			0.046
<37	549 (5.7)	497 (90.5)	52 (9.5)		505 (92.0)	44 (8.0)		528 (96.2)	21 (3.8)	
37–42	8762 (90.3)	8136 (92.9)	626 (7.1)		8318 (94.9)	444 (5.1)		8519 (97.2)	243 (2.8)	
≥42	392 (4.0)	350 (89.3)	42 (10.7)		363 (92.6)	29 (7.4)		374 (95.4)	18 (4.6)	
Unknown	51	45	6		48	3		50	1	
History of miscarriage, n (%)	2283 (23.4)	2079 (91.1)	204 (8.9)	0.002	2121 (92.9)	162 (7.1)	<0.001	2188 (95.8)	95 (4.2)	<0.001
Family history of allergy, n (%)	3079 (31.6)	2691 (87.4)	388 (12.6)	<0.001	2738 (88.9)	341 (11.1)	<0.001	2890 (93.9)	189 (6.1)	<0.001
Antibiotic use during pregnancy, n (%)	193 (2.0)	161 (83.4)	32 (16.6)	<0.001	161 (83.4)	32 (16.6)	<0.001	179 (92.7)	14 (7.3)	<0.001
Antibiotic use within the first year, n (%)	1625 (16.7)	1367 (84.1)	258 (15.9)	<0.001	1400 (86.2)	225 (13.8)	<0.001	1519 (93.5)	106 (6.5)	<0.001
Passive smoking, n (%)	4308 (44.2)	3889 (90.3)	419 (9.7)	<0.001	4056 (94.2)	252 (5.8)	0.043	4163 (96.6)	145 (3.4)	0.015
Decoration, n (%)	2816 (31.7)	2596 (92.2)	220 (7.8)	0.038	2635 (93.6)	181 (6.4)	0.003	2715 (96.4)	101 (3.6)	0.014
Unknown	870	765	105		827	43		848	22	
Having a pet since conception, n (%)				<0.001			<0.001			<0.001
Yes	1172 (12.0)	1056 (90.1)	116 (9.9)		1085 (92.6)	87 (7.4)		1117 (95.3)	55 (4.7)	
No	8582 (88.0)	7972 (92.9)	610 (7.1)		8149 (95.0)	433 (5.0)		8354 (97.3)	228 (2.7)	
Insecticide use, n (%)				<0.001			<0.001			0.001
Yes	3702 (38.0)	3362 (90.8)	340 (9.2)		3467 (93.7)	235 (6.3)		3569 (96.4)	133 (3.6)	
No	6052 (62.0)	5666 (93.6)	386 6.4)		5767 (95.3)	285 (4.7)		5902 (97.5)	150 (2.5)	
Dampness/mold, n (%)				<0.001			<0.001			<0.001
Yes	1447 (14.8)	1259 (87.0)	188 (13.0)		1321 (91.3)	126 (8.7)		1372 (94.8)	75 (5.2)	
No	7428 (76.2)	6967 (93.8)	461 (6.2)		7090 (95.4)	338 (4.6)		7249 (97.6)	179 (2.4)	
Unknown	879 (9.0)	802 (91.2)	77 (8.8)		823 (93.6)	56 (6.4)		850 (96.7)	29 (3.3)	
Traffic within 50 m, n (%)				0.013			<0.001			<0.001
Much	2499 (25.6)	2281 (91.3)	218 (8.7)		2311 (92.5)	188 (7.5)		2390 (95.6)	109 (4.4)	
Not much	4122 (42.3)	3824 (92.8)	298 (7.2)		3926 (95.2)	196 (4.8)		4019 (97.5)	103 (2.5)	
Less	3133 (32.1)	2923 (93.3)	210 (6.7)		2997 (95.7)	136 (4.3)		3062 (97.7)	71 (2.3)	
Frequency of park visits, n (%)				0.003			0.002			0.002
≤once/month	2272 (25.6)	2079 (91.5)	193 (8.5)		2120 (93.3)	152 (6.7)		2180 (96.0)	92 (4.0)	
2–3 times/month	2948 (33.2)	2736 (92.8)	212 (7.2)		2787 (94.5)	161 (5.5)		2866 (97.2)	82 (2.8)	
1–2 times/week	2341 (26.4)	2195 (93.8)	146 (6.2)		2230 (95.3)	111 (4.7)		2284 (97.6)	57 (2.4)	
3–5 times/week	938 (10.6)	886 (94.5)	52 (5.5)		894 (95.3)	44 (4.7)		912 (97.2)	26 (2.8)	
≥ once/day	385 (4.3)	367 (95.3)	18 (4.7)		376 (97.7)	9 (2.3)		381 (99.0)	4 (1.0)	
unknown	870	765	105		827	43		848	22	
Ambient temperature, °C, median (IQR)	26.1 (0.1)	26.1 (0.1)	26.1 (0.2)	0.120	26.1 (0.1)	26.1 (0.1)	>0.9	26.1 (0.1)	26.1 (0.1)	>0.9
Relative humidity, %, median (IQR)	82.3(0.5)	82.3 (0.5)	82.3 (0.5)	0.200	82.3 (0.5)	82.2 (0.3)	0.004	82.3 (0.5)	82.3 (0.5)	0.400

N: number; traffic within 50 m: traffic conditions within 50 m of the house; ^1^ *p*-value was calculated using Pearson’s chi-squared test, Mann–Whitney U test, or Fisher’s exact test, as appropriate.

**Table 2:** Association of frequency of exposure to insecticides with childhood asthma and wheezing.

Exposure Frequencies	Ever Asthma		Ever Wheezing		Current Wheezing	
	Crude Model	Adjusted Model	Crude Model	Adjusted Model	Crude Model	Adjusted Model
No exposure (n = 6052, 70.9%)	Reference	Reference	Reference	Reference	Reference	Reference
Only once (n = 477, 5.6%)	1.42 (1.00, 1.95)	1.15 (0.81, 1.61)	1.65 (1.13, 2.34)	1.39 (0.94, 2.00)	1.54 (0.91, 2.47)	1.26 (0.73, 2.04)
Once/half year (n = 877, 10.3%)	1.47 (1.14, 1.89)	1.21 (0.93, 1.56)	1.41 (1.04, 1.87)	1.07 (0.78, 1.45)	1.73 (1.18, 2.47)	1.35 (0.91, 1.95)
Once/quarter (n = 428, 5.0%)	1.47 (1.03, 2.05)	1.24 (0.86, 1.76)	1.53 (1.01, 2.22)	1.19 (0.78, 1.76)	1.43 (0.80, 2.37)	1.11 (0.62, 1.87)
Once/month (n = 442, 5.2%)	1.42 (1.00, 1.98)	1.18 (0.82, 1.67)	0.96 (0.58, 1.49)	0.77 (0.46, 1.22)	1.38 (0.77, 2.29)	1.15 (0.63, 1.94)
Once/week (n = 260, 3.1%)	2.51 (1.73, 3.56)	1.87 (1.26, 2.70)	1.87 (1.16, 2.88)	1.43 (0.86, 2.27)	2.24 (1.22, 3.79)	1.73 (0.92, 3.00)
OR for trend	1.14 (1.08, 1.20)	1.09 (1.03, 1.15)	1.09 (1.02, 1.16)	1.03 (0.96, 1.10)	1.15 (1.06, 1.24)	1.09 (1.00, 1.18)
*p* for trend	<0.001	0.001	0.007	0.380	<0.001	0.034

The data are presented as ORs and 95% CIs; n: number; crude model: univariable model; adjusted model: the model including child’s sex, gestational week, family history of allergy, antibiotic use during pregnancy, antibiotic use within child’s first year, exposure to passive smoking, mold, frequency of park visits, insecticide, and ambient temperature.

## Data Availability

Data will be made available upon reasonable request.

## Supplementary Materials

The following supporting information can be downloaded at https://www.mdpi.com/article/10.3390/toxics12060392/s1, Figure S1: Path diagrams of insecticide use and childhood asthma/wheezing. Green circle with triangle—exposure variable; blue circle with I— outcome variable; orange-red circles—causes of exposures; black circles filled with white and black lines—adjusted variables and causal paths; green lines—causal paths; orange-red lines—biasing paths. Table S1: The values of the variance inflation factor (VIF) in the adjusted models. Table S2: A comparison of the results of binomial generalized linear models (GLMs) with a logit link and mixed-effect regression models (MERMs), with districts as random effects.
