# Supplementary material for: Associations of Insecticide Exposure with Childhood Asthma and Wheezing: A Population-Based Cross-Sectional Study in Sanya, China
Source: Toxics. 2024 May 27;12(6):392. doi: 10.3390/toxics12060392 (PMC11209441; doi:10.3390/toxics12060392)
Supplement: Supplementary file 1 [file toxics-12-00392-s001.zip › toxics-3007193-supplementary.pdf]

Associations of Insecticide Exposure with Childhood Asthma and Wheezing: A Population-Based Cross-Sectional Study in Sanya, China

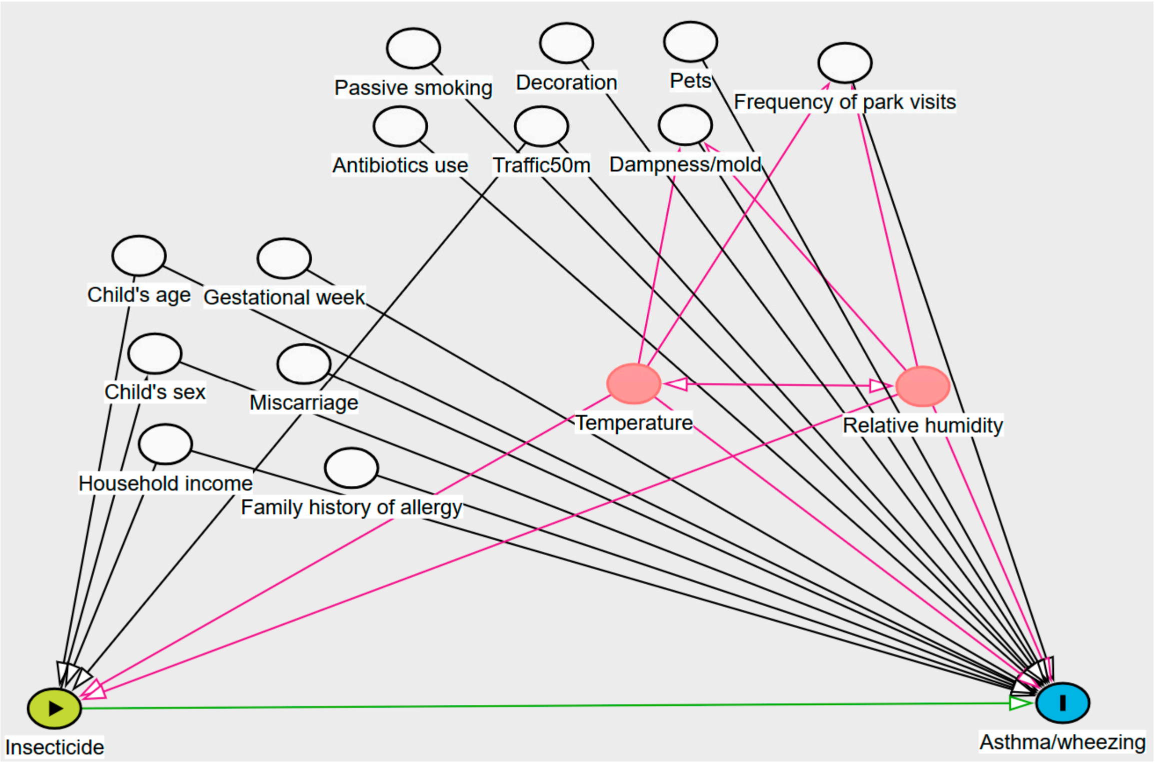

Figure S1. Path diagrams of insecticide use and childhood asthma/wheezing. Green circle with triangle, exposure variable; blue circle with I, outcome variable; orange-red circles, ancestors of exposures; black circles filled with white and black lines, adjusted variables and causal paths; green lines, causal paths; orange-red lines, biasing paths.

Table S1 The values of variance inflation factor (VIF) in the adjusted models

Variables	Ever asthma	Ever wheezing	Current wheezing
Insecticide	1.03	1.04	1.04
Child's sex	1.01	1.01	1.01
Gestational week	1.07	1.09	1.06
Family history of allergy	1.04	1.03	1.04
Antibiotics use during pregnancy	1.03	1.03	1.04
Antibiotics use within the first year	1.05	1.04	1.06
Passive smoking	1.05	1.05	1.05
Dampness/mold	1.08	1.08	1.09
Frequency of park visits	1.20	1.19	1.15
Ambient temperature	1.07	1.05	1.04
Mean VIF	1.06	1.06	1.06

The adjusted models: the model with the lowest AIC where child's sex, gestational week, family history of allergy, antibiotics use during pregnancy, and antibiotics use within the first year of child, exposures of passive smoking, mold, frequency of park visits, insecticide, and ambient temperature were included.

Table S2 Comparison results of binomial generalized linear models (GLM) with a logit link and mixed-effect regression models (MERM) with districts as random effects

**Variables**	**GLM (presented)**	**MERM (districts)**
	**OR (95% CI)**	**OR (95% CI)**
Total exposure		
Ever asthma	1.18 (1.00, 1.38)	1.18 (1.01, 1.39)
Ever wheezing	1.08 (0.89, 1.30)	1.09 (0.91, 1.32)
Current wheezing	1.15 (0.90, 1.47)	1.15 (0.90, 1.47)
Outdoor exposure		
Ever asthma	1.24 (1.03, 1.50)	1.24 (1.02, 1.50)
Ever wheezing	1.27 (1.03, 1.57)	1.26 (1.02, 1.56)
Current wheezing	1.38 (1.04, 1.81)	1.38 (1.04, 1.82)
Indoor exposure		
Ever asthma	1.12 (0.91, 1.38)	1.13 (0.91, 1.39)
Ever wheezing	0.82 (0.62, 1.07)	0.86 (0.65, 1.14)
Current wheezing	0.88 (0.61, 1.24)	0.87 (0.61, 1.24)

OR: odds ratio; CI: confidence intervals.
